# Genome-wide meta-analysis of QTL for morphological related traits of flag leaf in bread wheat

**DOI:** 10.1371/journal.pone.0276602

**Published:** 2022-10-24

**Authors:** Binbin Du, Jia Wu, Md. Samiul Islam, Chaoyue Sun, Baowei Lu, Peipei Wei, Dong Liu, Cunwu Chen

**Affiliations:** 1 College of Biotechnology and Pharmaceutical Engineering, West Anhui University, Lu’an, China; 2 Department of Plant Pathology, College of Plant Science and Technology and the Key Lab of Crop Disease Monitoring & Safety Control in Hubei Province, Huazhong Agricultural University, Wuhan, China; CSIR - Institute of Himalayan Bioresource Technology, India, INDIA

## Abstract

Flag leaf is an important organ for photosynthesis of wheat plants, and a key factor affecting wheat yield. In this study, quantitative trait loci (QTL) for flag leaf morphological traits in wheat reported since 2010 were collected to investigate the genetic mechanism of these traits. Integration of 304 QTLs from various mapping populations into a high-density consensus map composed of various types of molecular markers as well as QTL meta-analysis discovered 55 meta-QTLs (MQTL) controlling morphological traits of flag leaves, of which 10 MQTLs were confirmed by GWAS. Four high-confidence MQTLs (*MQTL-1*, *MQTL-11*, *MQTL-13*, and *MQTL-52*) were screened out from 55 MQTLs, with an average confidence interval of 0.82 cM and a physical distance of 9.4 Mb, according to the definition of hcMQTL. Ten wheat orthologs from rice (7) and *Arabidopsis* (3) that regulated leaf angle, development and morphogenesis traits were identified in the hcMQTL region using comparative genomics, and were speculated to be potential candidate genes regulating flag leaf morphological traits in wheat. The results from this study provides valuable information for fine mapping and molecular markers assisted selection to improve morphological characters in wheat flag leaf.

## Introduction

Wheat is one of the world’s three major crops, providing approximately a quarter of food for human. The continuous increase of wheat yield is crucial to meet the challenge of increasing food consumption [1]. Increasing planting density by improving the plant architecture of wheat on limited land is an effective strategy to increase yield [2]. In crops, canopy leaves, especially flag leaf, are the main source of dry matter accumulation in the grain filling stage [3, 4], and flag leaf provide 41–43% of carbohydrates for grain filling [5]. Therefore, optimizing the morphological structure of flag leaves is a suitable method to improve plant architecture, photosynthetic efficiency and yield.

Wheat flag leaf morphological traits are quantitative traits influenced by many environmental factors and controlled by multiple genes [6–8]. Many genes and QTLs that control leaf size and angle have been reported in rice [9–13]. For example, the mutation of *OsDWARF* gene resulted in the defect of brassinosteroid synthesis, which led to the reduction of plant height and the upright leaves [14]. In maize, Ku et al. [15] detected a major QTL *qLA2* controlling the angle of flag leaf on chromosome 2. Tian et al. [16] found that *UPA1* and *UPA2* genes can increase the planting density by regulating the leaf angle of plants, thus increasing the maize yield. QTLs for flag leaf length, width, area and angle have been identified on 21 chromosomes in wheat [17–22]. For example, Liu et al. [19] detected three major QTLs on 3D, 7B and 7D for flag leaf angle. Liu et al. [23] found that *TaSPL8* regulated leaf development by influencing auxin signal and brassinolide biosynthesis pathway, and affected flag leaf angle in wheat. Wang et al. [24] introduced the chromosome 1P of the wild related species *Agropyron cristatum* into common wheat to significantly reduce plant height and leaf size, thereby improving plant architecture and achieving dense planting.

Currently, many QTLs for flag leaf morphological related traits in wheat have been identified in previous studies. In order to make more effective use of the QTL for flag leaf morphological traits in wheat breeding, and deeply understand the genetic mechanism underlying flag leaf morphological traits, it is necessary to comprehensively analyze these QTLs to identify stable major genetic loci in wheat. QTL meta-analysis has been shown to be an effective method for integrating QTLs from various experiments onto a consensus map, narrowing QTL confidence intervals, and identifying reliable and stable meta-QTLs (MQTL) [25]. This method has been widely used in different crops for various traits, such as nematode resistance in soybean [26], yield under drought conditions in rice [27], yield and quality traits in cotton [28], yield in maize [29], and yield, nitrogen use efficiency, quality traits, disease resistance and abiotic stress tolerance in wheat [30–37].

With the development of high-throughput SNP sequencing technology, QTL mapping for complex quantitative traits based on natural populations using genome-wide association studies (GWAS) has been widely applied in rice [38], maize [39], wheat [40] and barley [41]. In addition, certain important QTLs have been identified by cross-validation based on the results of GWAS and linkage analysis in previous studies [42, 43]. These studies indicated that the QTL location information identified by GWAS can effectively verify important QTLs, so that key genomic regions and candidate genes controlling important quantitative traits can be mined.

To date, QTL meta-analysis for flag leaf morphological traits has not been reported in wheat. In this study, QTL meta-analysis was performed based on QTL for flag leaf morphological traits published since 2010, and GWAS was used to further validate the MQTL. Comparative genomics was used to identify wheat orthologs from rice and *Arabidopsis thaliana* to discover genomic regions and important candidate genes affecting flag leaf morphology in wheat. The aim of this study was to better understand the genetic mechanism underlying flag leaf morphological traits, and to provide useful information for genetic improvement of plant architecture and yield potential in wheat.

## Materials and methods

### Collection of QTL for wheat flag leaf morphological traits

Using public databases such as China National Knowledge Infrastructure (CNKI, https://www.cnki.net/), National Center for Biotechnology Information (NCBI, https://www.ncbi.nlm.nih.gov/) and Google Scholar (https://scholar.google.com/), 26 papers about QTL mapping for flag leaf length, width, area, length-width ratio and angle in wheat from 2010 to concerned year were collected [17–22, 44–63]. The information including population type and number, molecular marker type, LOD value, contribution rate and confidence interval was summarized in Table 1. Twenty papers were set aside for analysis because QTL flanking markers identified in some studies were not integrated into the consensus map.

**Table 1:** QTL reported for flag leaf morphological traits in wheat.

Reference	Population	Population type	Population size	Trait	Num. of QTL	Marker type
[17]	Yanda1817×Beinong6	RIL	269	FLL, FLW, FLA, FLANG	48	SNP, SSR
[18]	**Harry×Wesley**	**RIL**	**204**	**FLL, FLW, FLA**	**21**	**GBS**
[19]	ND3331×Zang1817	RIL	213	FLL, FLW, FLA, FLANG	23	SSR
[20]	**H461 × CM107**	**RIL**	**200**	**FLL**	**3**	**DArT**
[21]	**20828×Chuannong 16**	**RIL**	**199**	**FLL, FLW, FLA, FLWR, FLANG**	**122**	**55KSNP, SSR**
[22]	**20828×SY95-71**	**RIL**	**128**	**FLL, FLW, FLA, FLWR, FLANG**	**86**	**55KSNP**
[44]	Wangshuibai×Mianyang 99–323	NILs	132	FLW	1	SSR
	Wangshuibai×PH691		125			
[45]	Xiaoyan81×Xinong1376	RIL	236	FLL, FLW, FLA	31	SSR
[46]	Kenong9204×Jing411	RIL	188	FLL, FLW, FLA	38	SSR, DArT, STS, SRAP
[47]	Hanxuan10×Lumai14	DH	150	FLL, FLW, FLA	12	SSR
[48]	Ningchun4×Ningchun27	RIL	128	FLL, FLW	16	SSR
[49]	H461 × CN16	RIL	188	FLL, FLW, FLWR	16	90KSNP
[50]	Zhou8425B×Xiaoyan81	RIL	102	FLL, FLW, FLA	22	SNP, SSR
[51]	Ningchun4×Drasdale	RIL	148	FLL, FLW, FLA	22	SSR
[52]	Longjian 19×Q9086	RIL	120	FLL, FLW, FLA, FLWR	55	SSR
[53]	Nongda3338×Jingdong6	DH	216	FLL, FLW, FLA	40	SSR
[54]	Shanghai3×Catbird, Naxos	RIL	137	FLL, FLW	4	SSR
[55]	Weimai8×Luohan2	RIL	179	FLL, FLW, FLA	31	DArT
	Weimai8×Yannong19		175			
	Weimai8×Jimai20		172			
[56]	WL711×C306	RIL	206	FLL, FLW, FLA	7	SSR
[57]	Yanzhan 1 ×Cayazheda 29,	RIL	82	FLL, FLW, FLA	43	90KSNP
	Yanzhan 1 ×Yunnanxiaomai,		98			
	Yanzhan 1 ×Yutiandaomai,		93			
	Yanzhan 1 ×Hussar"		97			
[58]	**AS985472×Sumai 3**	**RIL**	**94**	**FLL, FLW**	**3**	**DArT**
[59]	Lumai 14×Jing 411	IL(BC_3_F_6_)	160	FLL, FLW, FLA	9,12	SSR
	Lumai 14×Shaanhan 8675		160			
[60]	Proteo×Chajia	RIL	97	FLL, FLW, FLA, FLWR	23	9KSNP, SSR
[61]	Xiaoyan81×Xinong1376	RIL	120	FLL	2	90KSNP
[62]	**EGA Wylie×Sumai 3**	**RIL**	**92**	**FLW**	**6**	**DArT**
[63]	Jingdong8×Aikang58	RIL	207	FLL, FLW, FLA, FLWR	10	SSR

^#^Bold font indicated that the study was not included in MQTL analysis.

FLL flag leaf length, FLW flag leaf width, FLA flag leaf area, FLWR flag length-width ratio, FLAG flag leaf angle.

### Integration of QTL for flag leaf morphological traits in wheat

In this study, the high-density map developed by Venske et al. [64] was used as the consensus map, which mainly includes three types of markers: SNP, DArT and SSR markers. SNP markers were derived from SNP array and genotyping-by-sequencing (GBS) [65, 66]. SSR markers came from three genetic maps (Wheat, Consensus SSR 2004, Wheat Composite 2004 and Wheat Synthetic × OPATA) in https://wheat.pw.usda.gov/GG3/ [67, 68]. The diversity Array technology (DArT) marker was derived from the wheat consensus map 4.0 integrated by more than 100 genetic maps. According to the LOD value, phenotypic variation explained (PVE), confidence interval and position of QTL, the QTL for the target trait was mapped to the consensus map by using BioMercator v4.2 software [69], and the principle that the flanking marker of QTL interval corresponds to the consensus map interval was followed. Before mapping to the consensus map, the 95% confidence intervals (CI) of QTL identified in different studies were inferred by using the following formulas: (1) C.I. = 530 / (N×PVE); (2) C.I. = 163 / (N×PVE); (3) C.I. = 287 / (N×PVE), C.I. is the confidence interval of QTL, N is the size of mapping population, the value 530, 163 and 287 are specific population constants calculated by different simulations, formula (1), (2) and (3) is suitable for F_2_ and backcross population, recombinant inbred line (RIL) population, and double haploid (DH) population, respectively [26, 70]. Details of these initial QTLs are listed in S1 Table.

### QTL meta-analysis and verification by GWAS

QTL meta-analysis for flag leaf morphological traits in wheat was carried out by using BioMercator v4.2 software. According to the number of QTL on each chromosome, two different analysis methods were used. When the QTL count on each chromosome is less than 10, MQTL is calculated for n independent QTLs by the method of Goffinet et al. [25]. Among the five models of 1, 2, 3, 4 and N, the lowest Akaike information criterion (AIC) value is considered as the best fitting model. When the QTL count on each chromosome exceeds 10, the method of Veyrieras et al. [71] is selected to determine the best QTL model based on AIC, AICc, AIC3, bayesian information criterion (BIC) and average weight of evidence (AWE), and the model with the lowest value of the selection criterion was used to determine MQTL.

All the flanking markers sequences of MQTL were BLASTed against the wheat Chinese spring reference genome sequence (RefSeq v1.0) to obtain the physical position of MQTL. Ten papers published in the past five years on the genome-wide association studies of flag leaf morphology in wheat were collected (Table 2), and the physical location of the MTA (maker-trait-association) in these studies was used to verify the accuracy of the MQTL region.

**Table 2:** The GWAS studies on flag leaf morphological traits used in this study.

No	Source of genotype	Population size	Trait	Marker type/number	Number of MTA^a^	Environment	Reference
1	Yellow and Huai River Valleys Wheat Zone	166	FLL, FLW	SNP/326570	13	Anyang, Suixi, Shijiazhuang, China	[40]
2	Yellow and Huai Valley of China	163	FLL, FLWR, FLW	SNP/20689	495	Zhumadian, Yuanyang, Zhengzhou, China	[60]
3	Chinese landraces and six landrace-derivatives	723	FLA, FLL, FLWR, FLW	DArT-seq/52303	14	Ya’an, Wenjiang, Chongzhou, China	[72]
4	Yellow and Huai Valley of China	163	FLAG	SNP/20689	86	Zhengzhou, Anyang, Zhumadian, China	[73]
5	Yellow and Huai Winter Wheat Region	197	FLL, FLW, FLAG	SNP/369869	145	Zhengzhou, China	[74]
6	Indian Spring Wheat	404	FLL	SNP/14160	3	Karnal, Bhavnagar, India	[75]
7	Yellow and Huai Valley of China	197	FLL, FLW, FLA, FLAG, FLWR	SNP/339266	439	Zhengzhou, Shangqiu, Zhumadian, China	[76]
8	Chinese winter wheat cultivars and non-Chinese parental lines	543	FLL, FLW, FLA, FLAG	SNP/11140	114	Baoding, Cangzhou, Xingtai, China	[77]
9	Chinese winter wheat	319	FLL, FLW	SNP/22905	39	Wuhan, China	[78]

^a^ Marker-trait association number (MTA) detected in previous GWAS studies.

FLL flag leaf length, FLW flag leaf width, FLA flag leaf area, FLWR flag length-width ratio, FLAG flag leaf angle.

### Mining candidate genes based on homology

According to the standard of mining highly reliable MQTLs by Venske et al. [71], MQTL with physical distance less than 20 Mb, genetic distance less than 1 cM and at least five overlapping QTLs were further selected as high confidence MQTL (hcMQTL). Combining with the genome annotation (https://wheat-urgi.versailles.inra.fr/seq-repository/annotations), the genes in the physical region of hcMQTL were analyzed, and the wheat orthologs in the physical region of hcMQTL were identified based on the genes related to flag leaf morphological traits of rice and *Arabidopsis thaliana* in Ensembl plant database (http://plants.ensembl.org/).

## Results

### QTL integration for flag leaf morphological traits in wheat

**Fig 1:**
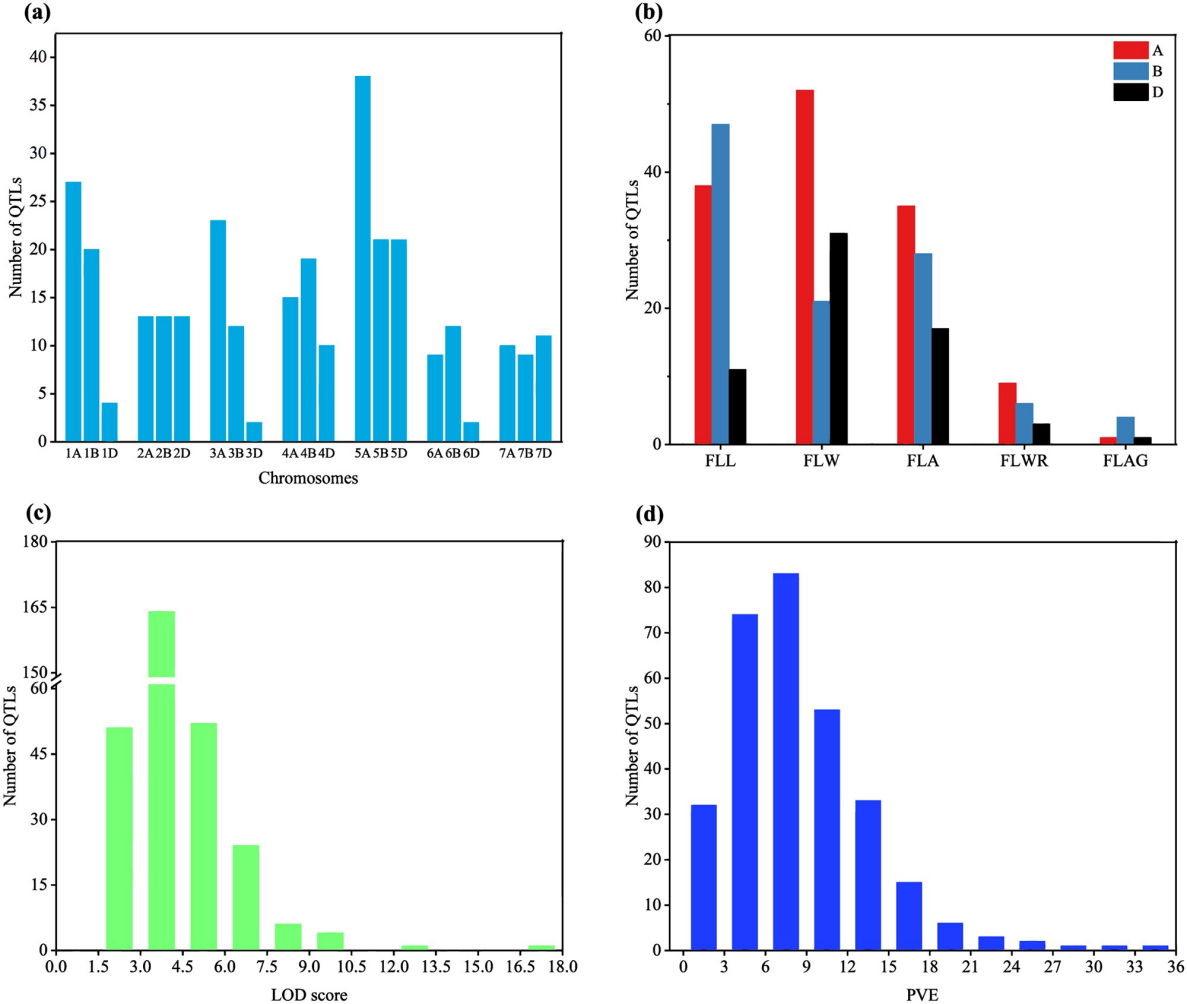
The information of QTL for wheat flag leaf morphological traits in previous QTL mapping studies. QTL distribution (a) on chromosomes of seven homoeologous groups, (b) for five flag leaf morphological traits, (c) according to the LOD value, and (e) according to the PVE value. *FLL* flag leaf length, *FLW* flag leaf width, *FLA* flag leaf area, *FLWR* flag length-width ratio, *FLAG* flag leaf angle.

A total of 465 QTLs related to flag leaf morphological traits were identified in the 20 papers published since 2010, covering 26 different mapping populations, among which 304 QTLs were projected into the consensus map. The number of QTL on each chromosome ranged from 2 on 3D to 38 on 5A, and the average number of QTLs on each chromosome was 14. Among them, 44.4% QTLs were distributed on A genome, 34.8% on B genome and 20.8% on D genome (Fig 1a). A total of 96 (31.6%), 104 (34.2%) and 80 QTLs (26.3%) were associated with flag leaf length, flag leaf width, and flag leaf area, respectively. Only 18 (5.9%) QTLs for flag leaf length-width ratio and 6 (2.0%) QTLs for flag leaf angle were identified (Fig 1b). The LOD score of individual QTLs ranged from 2.0 to 18.0, 54.28% of QTLs showed LOD scores from 3 to 4.5 (Fig 1c). The PVE range of individual QTL was 0.68–33.96%, and the PVE of 51.64% QTLs was within 3–9% (Fig 1d).

### QTL meta-analysis for flag leaf morphological traits in wheat

**Fig 2:**
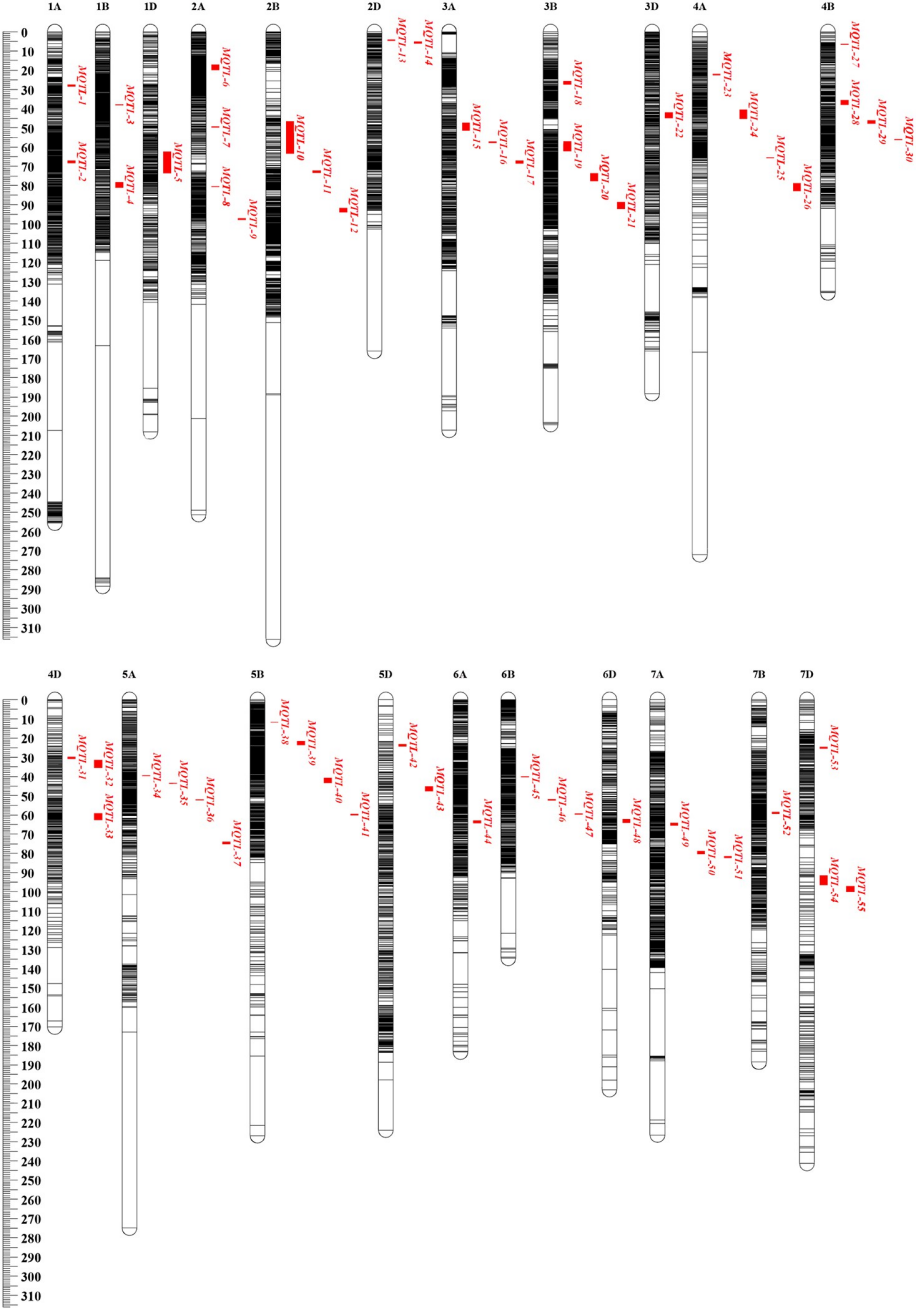
Distribution of 55 MQTLs on consensus map. Genetic distance scale in centiMorgan (cM) was placed at left margin. The horizontal bars in the genetic map represented the position of the markers.

**Fig 3:**
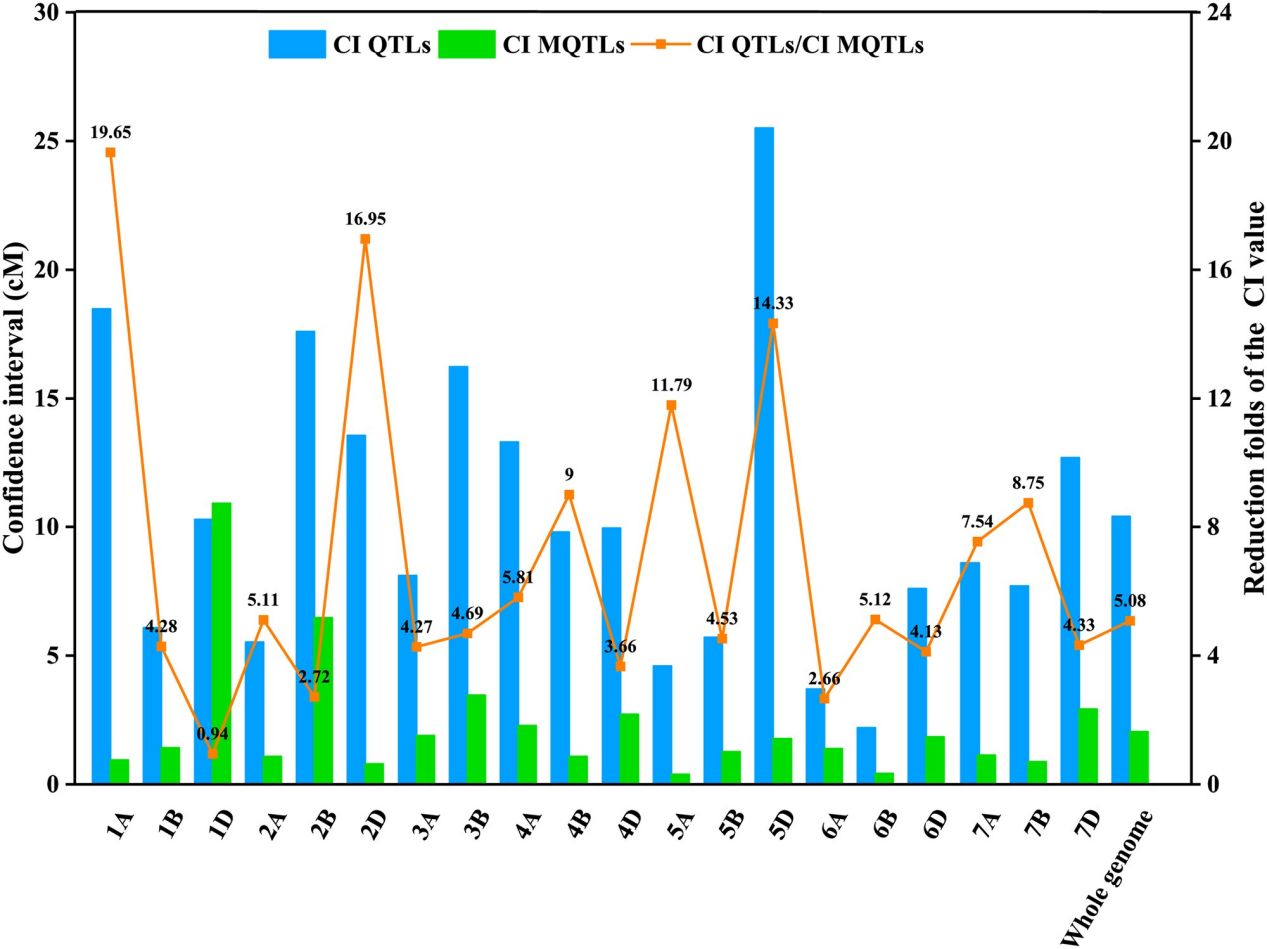
Comparison of confidence interval (CI) between initial QTLs (blue bar) and meta-QTLs (green bar).

A total of 304 QTLs were mapped to the consensus map, of which 275 QTLs were integrated into 55 MQTLs by meta-analysis, the remaining 29 QTLs were not integrated because they did not overlap with the above MQTLs (Table 3). These MQTLs were distributed on all chromosomes, with the number of MQTLs varying from one to four on each chromosome (Fig 2). The confidence interval of MQTL ranged from 0.06 to 16.45 cM, with the average interval size of 2.05 cM, which was 5.08-fold smaller than the initial QTL interval (Fig 3), the physical position interval ranged from 0.4 to 459.1 Mb, with the average physical distance of 66.5 Mb (Table 3).

**Table 3:** Meta-analysis of QTL for morphological traits of flag leaf in wheat.

MQTL	Chr	Position/cM	Confidence interval/cM	Flanking marker	Physical interval/Mb	Num. of QTL	Trait
*MQTL-1*	1A	28.11	27.64–28.38	*wsnp_Ex_c57982_59470152—wPt-7014*	8.3–19.8	9	FLL, FLW, FLA
*MQTL-2*		67.43	67.11–68.25	*1000535–2280626*	472.0–480.6	4	FLW, FLA
*MQTL-3*	1B	38	37.94–38.06	*3020845–1233770*	25.0–124.0	14	FLL, FLW, FLA, FLWR, FLANG
*MQTL-4*		79.72	78.36–81.08	*3023037–1042679*	658.7–661.4	4	FLW, FLA
*MQTL-5*	1D	67.99	62.54–73.45	*barc119_2–1229828*	367.4–435.5	3	FLW, FLA
*MQTL-6*	2A	18.5	17.11–19.88	*1151641–3958547*	89.6–101.0	2	FLL, FLA
*MQTL-7*		49.67	49.39–49.79	*CAP12_rep_c4192_354—BS00065276_51*	32.9–33.3	4	FLW, FLWR
*MQTL-8*		80.58	80.38–80.78	*wsnp_Ex_rep_c66448_64683704—wsnp_Ex_c20370_29434410*	84.9–93.9	3	FLL, FLW, FLA
*MQTL-9*		97.51	97.14–97.87	*Excalibur_c42512_584—BS00110386_51*	685.5–718.8	4	FLW, FLA
*MQTL-10*	2B	55	46.77–63.22	*RAC875_rep_c109471_154—RAC875_c38003_164*	4.4–23.5	4	FLL, FLA
*MQTL-11*		72.79	72.29–73.29	*Kukri_c11809_824—wsnp_Ra_rep_c106119_89961852*	15.7–28.4	5	FLL, FLW, FLA, FLWR
*MQTL-12*		92.89	91.91–93.87	*985860–3021999*	53.5–79.3	4	FLL, FLW, FLA
*MQTL-13*	2D	4.43	4.09–4.76	*3027483—Kukri_c77179_54*	2.5–10.5	9	FLL, FLW, FLA
*MQTL-14*		5.65	5.19–6.12	*Xwmc087—Xwmc453a*	56.8–70.1	3	FLW, FLA
*MQTL-15*	3A	49.35	47.43–51.27	*1112004—wsnp_Ex_c4069_7355431*	5.3–36.2	3	FLL, FLW
*MQTL-16*		57.47	57.28–57.66	*RAC875_c75448_80—Ku_c61039_98*	600.9–605.8	3	FLW, FLA
*MQTL-17*		67.74	67.0–68.48	*wsnp_Ex_c13452_21183096—BS00091002_51*	648.0–666.3	16	FLL, FLW, FLA, FLWR
*MQTL-18*	3B	26.63	25.83–27.43	*Kukri_c1771_715—BS00047114_51*	0.2–32.3	3	FLL, FLW, FLA
*MQTL-19*		59.59	57.07–62.11	*CAP8_c8651_206—wsnp_RFL_Contig3845_4190041*	40.3–47.7	3	FLL, FLW
*MQTL-20*		75.65	73.71–77.58	*1076556—Xbarc206*	667.8–700.9	3	FLW, FLA
*MQTL-21*		90.47	88.81–92.12	*Excalibur_c33274_498–977833*	704.5–749.8	3	FLL, FLW
*MQTL-22*	3D	43.5	42.11–44.88	*1061456–1229016*	62.1–179.3	2	FLW, FLWR
*MQTL-23*	4A	22.21	21.96–22.45	*1200937–1137855*	606.6–614.5	5	FLL, FLW, FLA
*MQTL-24*		43	40.73–45.26	*TA005380–0966—wsnp_Ex_rep_c104448_89161562*	596.8–605.0	2	FLW, FLA
*MQTL-25*		65.59	65.46–65.71	*3956825–1102806*	718.9–732.5	5	FLL, FLA
*MQTL-26*		80.82	78.87–82.77	*Xbcd130b–Xbarc78*	698.0–723.8	2	FLL, FLA
*MQTL-27*	4B	6.55	6.48–6.62	*wPt—5559–1863050*	2.3–2.7	3	FLL, FLA
*MQTL-28*		36.88	35.72–38.04	*Xfba41—wsnp_CAP7_c1723_854530*	20.6–21.6	6	FLL, FLW, FLA, FLWR
*MQTL-29*		46.94	46.24–47.65	*3946005—tPt—5342*	50.4–102.5	4	FLL, FLA, FLWR
*MQTL-30*		56.09	55.85–56.33	*Ku_c462_1417–1005100*	546.0–589.8	5	FLL, FLA, FLWR
*MQTL-31*	4D	30.02	29.82–30.71	*Xwmc473–984589*	11.5–227.2	3	FLL, FLW, FLANG
*MQTL-32*		33.53	31.63–35.43	*Xwmc182–1094332*	16.1–475.2	3	FLW, FLWR
*MQTL-33*		60.89	59.15–62.62	*993587—Ex_c41034_812*	209.2–380.2	4	FLL, FLW
*MQTL-34*	5A	39.63	39.57–39.68	*1371675–1212851*	253.6–306.5	16	FLL, FLW, FLA, FLWR
*MQTL-35*		43.6	43.47–43.73	*2294383–1034204*	494.5–503.0	5	FLL
*MQTL-36*		52.11	52.01–52.22	*992780—Excalibur_c41710_417*	555.2–594.1	13	FLL, FLW
*MQTL-37*		74.62	74.13–75.11	*Excalibur_c1954_930—BobWhite_c1763_558*	680.5–681.9	4	FLL, FLW, FLA, FLWR
*MQTL-38*	5B	11.85	11.82–11.88	*tPt—4875–1027318*	355.2–437.8	6	FLL, FLW
*MQTL-39*		22.63	21.68–23.58	*3943315–4541468*	368.8–502.3	5	FLL
*MQTL-40*		42	40.76–43.23	*345245–1019684*	485.6–590.5	2	FLL, FLA
*MQTL-41*		59.93	59.63–60.22	*2332836–3029473*	678.0–678.6	8	FLL, FLW, FLA, FLANG
*MQTL-42*	5D	23.9	23.29–24.52	*Xpsr326b–Xwmc318*	58.1–235.5	11	FLL, FLW, FLA
*MQTL-43*		46.47	45.31–47.64	*BobWhite_c10764_251—TA004396–0640*	401.4–411.2	5	FLL, FLW, FLA
*MQTL-44*	6A	63.6	62.9–64.29	*wsnp_BE495143A_Ta_2_1–994392*	574.2–580.2	8	FLL, FLW, FLA
*MQTL-45*	6B	40.13	40.02–40.25	*wsnp_JD_c2355_3205824–978170*	68.5–196.5	4	FLL, FLA
*MQTL-46*		52.07	51.77–52.37	*996529—Xcdo507*	556.5–669.4	5	FLL, FLW, FLA
*MQTL-47*		59.56	59.34–59.79	*4991087—RFL_Contig799_2434*	686.4–707.9	2	FLL
*MQTL-48*	6D	63.11	62.19–64.03	*1099552–1037337*	39.5–447.1	2	FLW
*MQTL-49*	7A	64.91	64.29–65.53	*WMC283—BS00044694_51*	62.1–64.8	5	FLL, FLW, FLA
*MQTL-50*		79.5	78.75–80.25	*1047407–1074455*	76.4–452.3	2	FLA, FLWR
*MQTL-51*		82.02	81.68–82.36	*RAC875_rep_c105584_237—RAC875_c52124_90*	26.4–93.1	3	FLW, FLA, FLWR
*MQTL-52*	7B	59.08	58.64–59.52	*987864–978206*	583.5–588.9	8	FLL, FLW, FLA
*MQTL-53*	7D	25.09	24.65–25.53	*978017—Excalibur_c27950_459*	3.1–54.0	5	FLL, FLW, FLA
*MQTL-54*		93.95	91.49–96.42	*Xbcd707—Xbarc26*	174.4–386.4	4	FLW
*MQTL-55*		99.14	97.11–100.09	*Xwmc221—Xwg719*	364.6–413.7	2	FLL, FLW

FLL flag leaf length, FLW flag leaf width, FLA flag leaf area, FLWR flag length-width ratio, FLAG flag leaf angle.

### MQTL validation by GWAS

**Fig 4:**
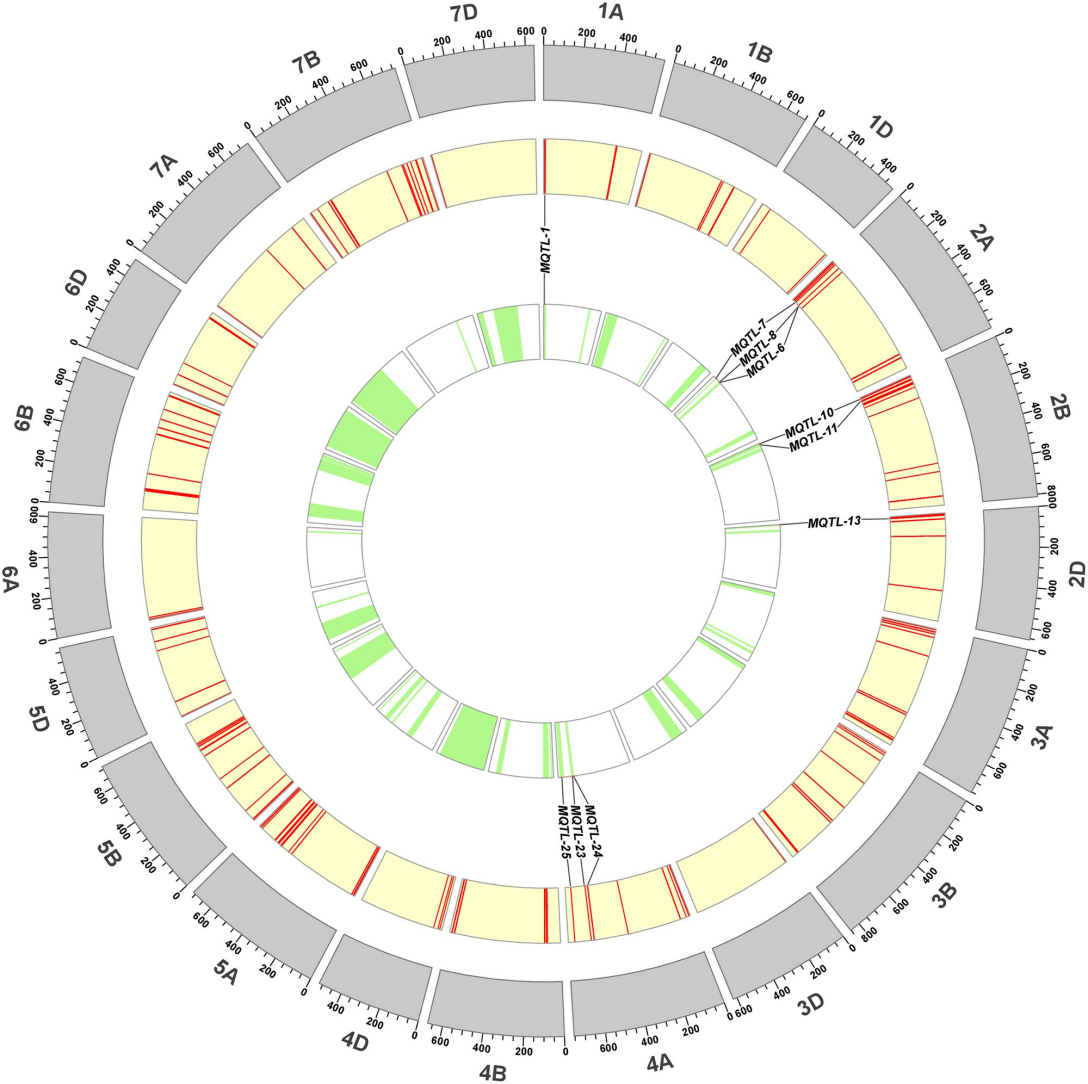
Validation of MQTL by MTAs on wheat flag leaf morphological traits from GWAS results published in recent years. The circles from inside to outside indicated the position of MQTL on the physical map, the position of MTA on the physical map and the physical map, respectively.

Among the 55 MQTLs, 25 (45.45%) MQTLs were mapped into physical region smaller than 20 Mb in the wheat reference genome (Table 3). To determine the accuracy of MQTL region, GWAS studies on wheat flag leaf morphology reported in the past five years were used to verify MQTL. Since the decay distance of the wheat linkage disequilibrium was about 5 Mb, those overlapping MQTLs within 5 Mb of MTA were considered to be co-located with MQTL. Ten of the 25 MQTLs were verified in at least one GWAS study and co-located with 45 MTAs (Fig 4). The number of MTAs co-located in each MQTL ranged from 1 to 22, in which *MQTL-10* co-located with 22 MTAs, followed by *MQTL-13* that co-located with 11 MTAs. In addition, clusters or nested distributions of MQTL were observed, such as *MQTL-6* (2A: 89.6–101.0 Mb) & *MQTL-8* (2A: 84.9–93.9 Mb) and *MQTL-23* (4A: 606.6–614.5 Mb) & *MQTL-24* (4A: 596.8–605.0 Mb) (Fig 4).

### Mining candidate genes based on homology within hcMQTL region

According to the definition of hcMQTL, *MQTL-1*, *MQTL-11*, *MQTL-13* and *MQTL-52* were eligible. These four MQTLs regulated multiple flag leaf traits with multi-effects, which indicated that they might have an important contribution to the regulation of flag leaf morphology. The mean confidence interval of the four hcMQTLs for *MQTL-1*, *MQTL-11*, *MQTL-13*, and *MQTL-52* was 0.82 cM. The physical distances for *MQTL-1*, *MQTL-11*, *MQTL-13*, and *MQTL-52* were 11.5 Mb, 12.7 Mb, 8 Mb, and 5.4 Mb, respectively, with the average physical distance of 9.4 Mb. The number of genes within the intervals were 453, 532, 380, and 149, respectively (S2 Table). In order to identify candidate genes related to leaf morphology among the four hcMQTLs, based on the Ensembl plant database (http://plants.ensembl.org/), 11 genes (six for rice and five for *Arabidopsis*) that regulate leaf morphology from the hcMQTL region were identified, of which, seven wheat orthologs for *Osmtd1*, three orthologs for *FRS7*, two orthologs for *Roc8*, and only one ortholog each for the other genes were found (Table 4).

**Table 4:** Eleven identified leaf morphology—Related genes of rice and *Arabidopsis thaliana* and their wheat orthologs in hcMQTLs region.

Gene ID	Gene Name	Function description	Species	Traits	Homologous gene ID in wheat	Corresponding MQTL region	Reference
*AT3G06250*	*FRS7*	FAR1-related sequence 7	*Arabidopsis*	flowering time, leaf growth	*TraesCS1A02G023000LC TraesCS1A02G031400LC TraesCS1A02G037300LC*	*MQTL-1*	[79]
*LOC_Os08g34258*	*Osmtd1*	Putative protease inhibitor I family protein, Control of plant architecture	Rice	tiller number, leaf angle	*TraesCS1A02G022100 TraesCS1A02G022200 TraesCS1A02G022300 TraesCS1A02G024800 TraesCS1A02G024900 TraesCS1A02G025000 TraesCS1A02G025300*	*MQTL-1*	[80]
*AT1G09700*	*HYL1*	dsRNA-binding domain-like superfamily protein	*Arabidopsis*	abscisic acid, auxin, and cytokinin	*TraesCS1A02G035900LC*	*MQTL-1*	[81]
*LOC_Os06g10600*	*Roc8*	Similar to Homeodomain protein HOX3	Rice	size of bulliform cells, lignin content	*TraesCS1A02G039400LC TraesCS1A02G039500LC*	*MQTL-1*	[82]
*LOC_Os01g15340*	*OsRAA1*	encodes a 12.0-kD protein	Rice	leaf, flower, and root development	*TraesCS1A02G039800LC*	*MQTL-1*	[83]
*AT1G07630*	*PLL5*	pol-like 5	*Arabidopsis*	leaf morphology	*TraesCS1A02G033700*	*MQTL-1*	[84]
*LOC_Os09g37400*	*OsSAUR45*	Small auxin-up RNA (SAUR), Auxin-responsive SAUR gene family member, Auxin synthesis and transport, Plant growth	Rice	plant height, primary root length, adventitious roots, leaf width and seed setting	*TraesCS2B02G045900LC*	*MQTL-11*	[85]
*LOC_Os05g11730*	*GSK2*	GSK3/SHAGGY-like kinase, Brassinosteroid signalin	Rice	plant height, leaf angle, and grain size	*TraesCS2B02G046300LC*	*MQTL-11*	[86]
*AT2G02560*	*CAND1*	cullin-associated and neddylation dissociated	*Arabidopsis*	flowering, fertility, dwarfism and leaf development	*TraesCS2B02G051000*	*MQTL-11*	[87]
*LOC_Os03g04680*	*SD37*	Cytochrome P450 protein CYP96B4, Growth regulation, Drought stress response	Rice	plant height, leaves, panicles, and seeds	*TraesCS2D02G005000LC*	*MQTL-13*	[88]
*AT5G61020*	*ECT3*	evolutionarily conserved C-terminal region 3	*Arabidopsis*	timing of leaf formation, leaf morphology	*TraesCS2D02G012200*	*MQTL-13*	[89]

## Discussion

QTL meta-analysis developed by Goffinet et al. [25] is a method for identifying consistent and stable QTLs and improving the accuracy of their genetic positions. The length, width, area, and angle of flag leaves are all important factors in determining wheat plant architecture and yield potential [5, 90–92]. Many genetic studies have been conducted to identify QTL for flag leaf morphological traits in wheat (Table 1). Most of the initial QTLs collected in this study were distributed on A genome, and the least on D genome, which was slightly different from the results of previous studies regarding the distribution of initial QTLs for wheat yield and related traits on the genome (the most QTLs were distributed on B genome, but the least on D genome) [36, 93], which might be due to the limited number of QTLs for flag leaf morphological traits, resulting in inconsistent results with previous studies. The less QTL on D genome may be related to the low-level polymorphism on D genome [94].

In this study, the maximum likelihood estimation method was used in meta-analysis in combination with the genetic locations of hundreds of QTLs for flag leaf morphological traits in wheat, and with consideration of population size and other QTL information, 275 of the 304 QTLs were mapped onto the consensus map and integrated into 55 MQTLs in wheat. Due to the pleiotropic effect of genes on flag leaf morphology in wheat, more than 90% (50/55) of the MQTLs were associated with at least two flag leaf morphological traits, and about 43.64% (24/55) of the MQTLs affected three or more flag leaf morphological traits simultaneously (Table 3).

After integrating QTLs by meta-analysis, the average confidence interval of MQTL was 2.05 cM, which was about 5.08-fold smaller than the average confidence interval (10.41 cM) of the initial individual QTL (Fig 3). Accordingly, the physical intervals of MQTL on chromosomes were further reduced, improving the accuracy of QTL mapping. The primary QTL mapping to fine mapping usually needs to increase molecular marker density [95] or construct fine mapping populations such as near-isogenic lines [96, 97]. In certain cases, QTL meta-analysis could replace or enhance these approaches. For example, *MQTL-52* was integrated by eight QTLs for flag leaf length, flag leaf width, and flag leaf area from two different populations and finally located within the interval of 58.64–59.52 cM on chromosome 7B, with the physical interval of 583.5–588.9 Mb, which was much smaller than the confidence interval of the initial QTL.

Compared with QTL linkage analysis mapping, linkage disequilibrium-based genome-wide association studies (GWAS) is another method for precisely locating genomic regions of quantitative traits. In previous studies, the results of wheat MQTL verification by GWAS have been reported [98, 99]. For example, Aduragbemi et al. [100] identified 51 MTA and 29 MQTLs co-located for leaf rust resistance loci using GWAS. Yang et al. [101] verified MQTL for wheat yield and yield-related traits using GWAS results published in recent years, and found that about 60% of MQTLs were co-located with MTA. In this study, based on the GWAS results of wheat flag leaf morphological traits published in recent years, 45 MTAs and 10 MQTLs were identified, which indicated that these genomic regions controlling flag leaf morphological traits might be less affected by the genetic background and environment. The 10 MQTLs verified by GWAS provided a basis for the accurate mining candidate genes that affect flag leaf morphology in wheat. Loffler et al. [102] proposed the criteria for selection of MQTL for use in breeding programs: the MQTL with confidence interval genetic distance less than 2 cM, no less than 4 initial QTLs from different studies with PVE> 10%. On this basis, we determined three potential MQTLs, *MQTL-1*, *MQTL-13* and *MQTL-25*, that could be used to improve wheat flag leaf morphological traits.

Flag leaf morphology is one of the important traits of plant architecture in wheat breeding. Moreover, previous studies reported the correlation between flag leaf morphology and plant structure traits such as plant height and tiller number [57, 78]. Hu et al. [57] revealed the genetic mechanism of yield-related traits in wheat using four RIL populations and found that plant height had a significant positive correlation with FLL and FLW, and QTL affecting both plant height and FLW were detected at 0–3.5 cM on chromosome 5A. In addition, Muhammad et al. [78] identified five SNP markers affecting PH, FLL and FLW simultaneously on chromosomes 1A, 3A, 3B, 5A, and 6B in natural populations of wheat. Some previously reported major QTLs and genes controlling plant height and tillering number in wheat were identified in the hcMQTL region in this study. The gene *Csl-1A* (chr1A:6.4 Mb) controlling the tiller number in wheat [103] was identified near MQTL-1. Saini et al. [36] collected QTLs for wheat yield and related traits in the past 20 years and identified 141 MQTLs, of which five MQTLs (*MQTL1A*.*5*, *MQTL2B*.*3*, *MQTL2B*.*4*, *MQTL2B*.*5*, and *MQTL2D*.*2*) affecting traits such as plant height and tillering number were located at approximately 5 Mb in the *MQTL-1*, *MQTL-11*, and *MQTL-13* regions. These examples indicate that these three hcMQTLs may carry some major genes that improve the plant architecture of wheat, such as plant height and tiller.

In cereal with large complex genomes, such as wheat, barley and maize, localization based on homologous cloning is an effective way to identify important genes associated with complex traits. With the wide application of high-throughput sequencing technology, many crops genome sequences have been published, which conduces to identify conserved genome regions and key genes in different crops. For example, the rice *OsLG1* gene encodes a SBP DNA binding protein, which affects the development of auricle and ligule [104], and the orthologous gene *TaSPL8* in wheat has also been found to have similar function in rice [23].

In this study, a total of 20 wheat orthologs were identified in four hcMQTLs, 10 of which were low-confidence, and the annotation information might be inaccurate. The remaining 10 wheat orthologs were potential candidate genes for regulating the leaf morphology in wheat, including 7 from rice genes and 3 from *Arabidopsis* genes (Table 4). In total, 7 wheat orthologs of the rice gene *Osmtd1* were located in the *MQTL-1* region, namely *TraesCS1A02G022100*, *TraesCS1A02G022200*, *TraesCS1A02G022300*, *TraesCS1A02G024800*, *TraesCS1A02G024900*, *TraesCS1A02G025000* and *TraesCS1A02G025300*. *Osmtd1* gene encodes a putative inhibitor I family protein regulating rice tillering and leaf angle [80]. Therefore, these seven wheat orthologs may be reliable candidates for regulating wheat leaf angle as the *Osmtd1* gene in rice.

The wheat ortholog of *Arabidopsis PLL5* gene, *TraesCS1A02G033700*, is located in the *MQTL-1* region and encodes a protein belonging to the phosphatase 2C family, which regulates leaf development. The mutant *pll5* has shorter, narrower and curlier leaves than the wild-type leaves [84]. Hence, it suggested that *TraesCS1A02G033700* is a credible candidate gene affecting leaf development in wheat. The *CAND1* gene encodes unmodified CUL1-interacting protein in *Arabidopsis*, and participates in many developmental pathways controlled by ubiquitin/proteasome-mediated degradation of protein [87]. The rosette leaves of *cand1* mutants are much smaller than that of wild-type plants and have a wavy morphology. The ortholog *TraesCS2B02G051000* of wheat located in *MQTL-11* region encodes CUL1-related NEDD8 dissociation protein, which might be a candidate gene affecting the wheat leaves morphology. The *ECT3* gene encodes the YTH domain protein in *Arabidopsis*, which has been previously proved to be related to leaf morphogenesis in *Arabidopsis* [89]. The wheat ortholog *TraesCS2D02G012200*, located in the *MQTL-13* region, might be a reliable candidate gene involved in the regulation of leaf development.

In conclusion, using the high-density integration map developed by Venske et al. [64] as the consensus map and QTL meta-analysis, we integrated the QTL for flag leaf morphological traits previously identified in wheat, and validated 10 MQTLs with GWAS information. Three potential MQTLs, *MQTL-1*, *MQTL-13* and *MQTL-25* that regulate flag leaf morphological traits were identified in this study. These MQTL flanking markers can be used for molecular marker assisted breeding to improve flag leaf morphological traits in wheat. Furthermore, using functional annotation information from genes within the hcMQTL interval and a comparative genomics strategy, ten wheat orthologs were identified as potential candidate genes affecting wheat flag leaf morphology, providing potential targets for fine mapping, and gene cloning.

## Supporting information

S1 Table Initial QTL information for QTL meta-analysis. (XLSX)

S2 Table Gene annotation information in hcMQTL region. (XLSX)
